# Low HbA2 Level in β-Thalassemia Trait

**DOI:** 10.4274/tjh.37891

**Published:** 2013-03-05

**Authors:** Şinasi ÖZSOYLU

**Affiliations:** 1 Retired Professor of Pediatrics, Hematology, and Hepatology; 2 Honorary Fellow of American Academy of Pediatrics; 3 Honorary Member of American Pediatric Society; 4 Fellow of Islamic World Academy of Sciences

**To the Editor**,

Dr. Köseler and her colleagues reported the presence of δ-thalassemia in 3 out of 12 patients carrying the β-thalassemia trait with low HbA2 in the recent issue of this journal without giving any explanations for the remaining 9 cases (2012; 29: 289-290) [1].

I wish that they would also look for the presence of α-thalassemia, at least in those 9 cases, because this seems to be the more prevalent type of thalassemia in our country, as first shown by us [2,3] and as further supported by Canatan et al. in this journal [4].

**Conflict of Interest Statement**

The authors of this paper have no conflicts of interest, including specific financial interests, relationships, and/ or affiliations relevant to the subject matter or materials included.

## REPLY

We are thankful for the valuable comments of Prof. Dr. Şinasi Özsoylu in regard to our letter entitled “HbA2-Yokoshima (delta 25(B7)Gly-Asp) and HbA2-Yialousa (delta 27(B9)Ala-Ser) in Turkey”, published in the Turkish Journal of Hematology (2012; 29: 289-290).

We reported in our letter the presence of the abnormal hemoglobin variants known as HbA2-Yokoshima and HbA2-Yialousa in Denizli Province in Turkey. Our letter was not concerned with delta-thalassemia. Since these delta variants could affect the value of HbA2 in beta-thalassemia carriers in laboratory diagnosis, we emphasized the importance of that effect.

Unfortunately, we have neither the data nor the observation and concern for the possible effect of alpha-thalassemia on the laboratory value of HbA2 in our letter.

**Aylin Köseler, Ayfer Atalay, Erol Ömer Atalay**
